# Levetiracetam versus carbamazepine monotherapy in the management of pediatric focal epilepsy: A systematic review and meta-analysis of randomized controlled trials

**DOI:** 10.1007/s00431-024-05768-0

**Published:** 2024-09-18

**Authors:** Jefferson Manoel Borges Martins, Paula Larissa Ferreira Vieira, Giovanni Gosch Berton, Vanessa Karlinski Vizentin, Rafael dos Santos Borges, Ana Livia Chaves Vieira, Celina Cláudia Israel Sefer, Aurimery Gomes Chermont

**Affiliations:** 1 Division of Medicine, Federal University of Pará, Belém, PA Brazil; 2 Department of Medicine, School of Medicine, University of Padua, Ospedale Civile St., 77, 35121 Padua, Veneto Italy; 3 School of Medicine, University of Passo Fundo, Passo Fundo, RS Brazil; 4 Department of Cardiovascular Diseases, Mayo Clinic, Rochester, MN USA; 5 Division of Medicine, Federal University of Minas Gerais, Belo Horizonte, MG Brazil; 6 Division of Medicine, Metropolitan University Center of the Amazon, Belém, PA Brazil

**Keywords:** Focal epilepsy, Pediatric, Levetiracetam, Carbamazepine, Monotherapy

## Abstract

Levetiracetam (LEV) and carbamazepine (CBZ) are effective monotherapies for focal epilepsy in children. However, the best drug remains controversial. Therefore, we performed a systematic review and meta-analysis comparing LEV and CBZ monotherapy in the management of pediatric focal epilepsy (PFE). We searched PubMed, Embase, and Cochrane databases for randomized controlled trials (RCTs) published until February 2024 comparing LEV and CBZ monotherapy in PFE. Statistical analysis was performed using R version 4.2.2, heterogeneity was assessed using I^2^ statistics, and the risk of bias was evaluated using the RoB-2 tool. Risk Ratios (RR) with *p* < 0.05 were considered significant. The outcomes of interest were seizure freedom, any adverse events, adverse events leading to treatment discontinuation, dermatologic adverse events, and the frequency of at least one seizure, defined as the proportion of patients experiencing one or more seizures during the treatment period. Four RCTs comprising 381 children with a mean age of 7.32 to 9.28 years were included, of whom 186 (48.8%) received LEV monotherapy. There was no significant difference between groups (RR: 1.15; 95% CI 0.88–1.50; *p* = 0.31; I^2^ = 90%) regarding seizure freedom. The frequency of at least one seizure (RR: 0.71; 95% CI 0.52–0.97; *p* = 0.03; I^2^ = 8%) and dermatologic adverse events (RR: 0.24; 95% CI 0.09–0.64; *p* < 0.01; I^2^ = 0%) were both significantly lower in the LEV group. There were no significant differences in the presence of any adverse events (RR: 0.58; 95% CI 0.33–1.01; *p* = 0.05; I^2^ = 36%) or adverse events leading to treatment discontinuation (RR: 0.67; 95% CI 0.13–3.42; *p* = 0.63; I^2^ = 30%).

*Conclusion*: In monotherapy, LEV was more advantageous than CBZ for PFE, with a lower frequency of seizures and fewer dermatological adverse events. However, both drugs are equally effective in achieving seizure freedom, adverse events without specification, and those that lead to treatment discontinuation. Our findings have important implications for clinical practice and decision-making in this condition.

**Supplementary Information:**

The online version contains supplementary material available at 10.1007/s00431-024-05768-0.

**Table:** 

**What is Known:**
• *Both LEV and CBZ are effective monotherapies for pediatric focal epilepsy.*
• *The use of LEV or CBZ monotherapy for the management of children with focal epilepsy remains controversial.*
**What is New:**
• *No significant differences were observed between the LEV and CBZ groups in terms of overall seizure freedom, safety, and tolerance.*
• *However, LEV resulted in a lower frequency of at least one seizure and fewer dermatological adverse events than CBZ.*

## Introduction

Pediatric epilepsy is a major health concern worldwide. Focal seizures, which arise in a specific area of the brain, constitute a prevalent subtype of seizures in the pediatric population. These seizures can have profound effects on a child’s cognitive and emotional development as well as their quality of life. Managing focal seizures in pediatric patients requires a multifaceted approach, often involving pharmacotherapy as the foundation treatment. The selection of appropriate antiepileptic drugs is crucial for achieving optimal seizure control while minimizing adverse effects and preserving neurodevelopmental outcomes [1, 2].

Carbamazepine (CBZ) is a long-standing treatment for focal seizures in pediatric patients. Its efficacy in seizure reduction and improvement of seizure control has been extensively documented in clinical trials and practice. As a first-line agent, the mechanism of action of CBZ involves blocking voltage-gated sodium channels, thereby stabilizing neuronal membranes, and reducing hyperexcitability. Despite its established efficacy, concerns regarding tolerability, particularly potential cognitive side effects and hepatic toxicity persist. These considerations underscore the necessity for the comparative evaluation of new antiepileptic agents [3].

In recent years, levetiracetam (LEV) has emerged as a promising alternative treatment for focal pediatric seizures. Although not entirely understood, its mechanism of action is thought to involve modulation of synaptic neurotransmitter release. Compared to traditional antiepileptic drugs, LEV offers several advantages, including a favorable pharmacokinetic profile, minimal drug interactions, and reduced risk of cognitive side effects. Studies have suggested comparable efficacy between LEV and CBZ, with some evidence pointing towards superior tolerability of LEV. However, comprehensive comparisons between these agents are essential to guide evidence-based treatment decisions in pediatric patients [4, 5].

Despite the availability of individual trials comparing LEV and CBZ in pediatric focal seizures, discrepancies in study design, patient characteristics, and outcomes raise the need for a systematic review and meta-analysis. This study aimed to fill this gap by meta-analyzing existing evidence from randomized controlled trials (RCTs) to comprehensively evaluate the comparative efficacy, safety, and tolerability of LEV and CBZ in pediatric focal epilepsy (PFE).

## Methods

### Eligibility criteria

We included studies that met the following eligibility criteria: (1) RCT; (2) comparing LEV with CBZ; (3) in the population with PFE; (4) reporting at least one of the outcomes of interest; and (5) published in the English language. Letters, editorials, reviews, studies without a control group, and studies with overlapping patient populations were excluded. In this case, only the study with the largest sample size was included.

### Search strategy and data extraction

We systematically searched PubMed, Embase, and the Cochrane Central Register of Controlled Trials for RCTs published until February 2024 using the following search terms: (children OR child OR childhood OR pediatric) AND (levetiracetam) AND (carbamazepine) AND (focal OR partial) AND (epilepsy OR epilepsies OR seizure OR seizures) AND (randomized OR randomised OR random). References from all included studies and reviews were manually searched. All identified articles were systematically assessed using the inclusion and exclusion criteria. Article selection (J.M.B.M. and P.L.F.V.) and data extraction (V.K.V. and J.M.B.M.) were independently performed by at least two reviewers. The extracted data included the first author’s name, country of origin, year of publication, study design, number of participants, patient demographic characteristics, interventions, duration of follow-up, and primary and secondary outcomes. Disagreements between authors were resolved by consensus. The study protocol was prospectively registered with PROSPERO on February 2024 (ID: CRD42024517575). This study was conducted according to the pre-registered protocol, despite the choice of statistical software and the change in the measure of effects from OR to RR to promote a straightforward interpretation.

### Outcomes

The primary outcome of interest was (1) seizure freedom, defined as the complete absence of seizures during the treatment period. Secondary outcomes included: (2) frequency of at least one seizure, indicating the proportion of patients experiencing one or more seizures during the treatment period; (3) any adverse events, capturing all reported side effects regardless of severity; (4) adverse events leading to treatment discontinuation, identifying cases where treatment was halted due to adverse reactions; and (5) dermatologic adverse events, focusing specifically on skin-related side effects experienced during the treatment period.

### Quality assessment

The Cochrane tool for assessing the risk of bias in randomized trials (RoB-2) was used for the quality assessment [6]. The risk of bias assessment was conducted by two independent authors (G.G.B. and J.M.B.M.), who evaluated the risk of bias across various domains, including randomization, allocation to intervention, adherence to intervention, handling of missing outcome data, measurement of outcome, and selection of reported results. We collected data on outcomes based on intention-to-treat analysis. The overall risk of bias was categorized as “low,” “some concerns,” or “high” for each domain, both across and within individual studies. Disagreements were resolved through consensus.

Two independent authors (R.S.B. and P.L.F.V.) followed the Grading of Recommendations, Assessment, Development, and Evaluation (GRADE) handbook guidelines to assess the level of certainty of the evidence in this meta-analysis, with categorizations ranging from very low to high [7]. Disagreements were resolved through discussions with a third author (J.M.B.M.).

Publication bias was analyzed with a funnel plot. Which plotted individual study weights against the point estimates. The Egger test was not performed, following the Cochrane guidelines, because of the limited number of studies included in this meta-analysis (*n* < 10) [8].

### Statistical analysis

This systematic review and meta-analysis was performed and reported according to the Cochrane Collaboration Handbook for Systematic Reviews of Interventions and the Preferred Reporting Items for Systematic Reviews and Meta-analysis (PRISMA) statement guidelines [8, 9]. All analyzed outcomes were dichotomous, and risk ratios (RR) with 95% confidence intervals (CI) were used to compare the treatment effects. Heterogeneity was assessed using I^2^ statistics and Cochran’s Q test. Outcomes were considered to have low heterogeneity if *p* > 0.10 and I^2^ < 25%, moderate heterogeneity if I^2^ was between 25 and 75%, and high heterogeneity if I^2^ > 75%. Statistical significance was set at *p* < 0.05.

We used the common (fixed) effect model with the Mantel–Haenszel method for outcomes with low heterogeneity. If moderate or high heterogeneity was present, we applied the random-effects model of DerSimonian and Laird [10]. Additionally, for outcomes with moderate or high heterogeneity, we performed sensitivity analyses using the Sidik-Jonkman-Hartung-Knapp method, as recommended in the Cochrane Handbook, Sect. 10–10-4–4 [8]. Statistical analyses were performed using R version 4.2.2 (The R Foundation for Statistical Computing) with ‘meta’ and ‘metafor’ packages, and RStudio version 2024.04.2 + 764 (RStudio Team) [11–13].

## Results

### Study selection and baseline characteristics

**Fig. 1:**
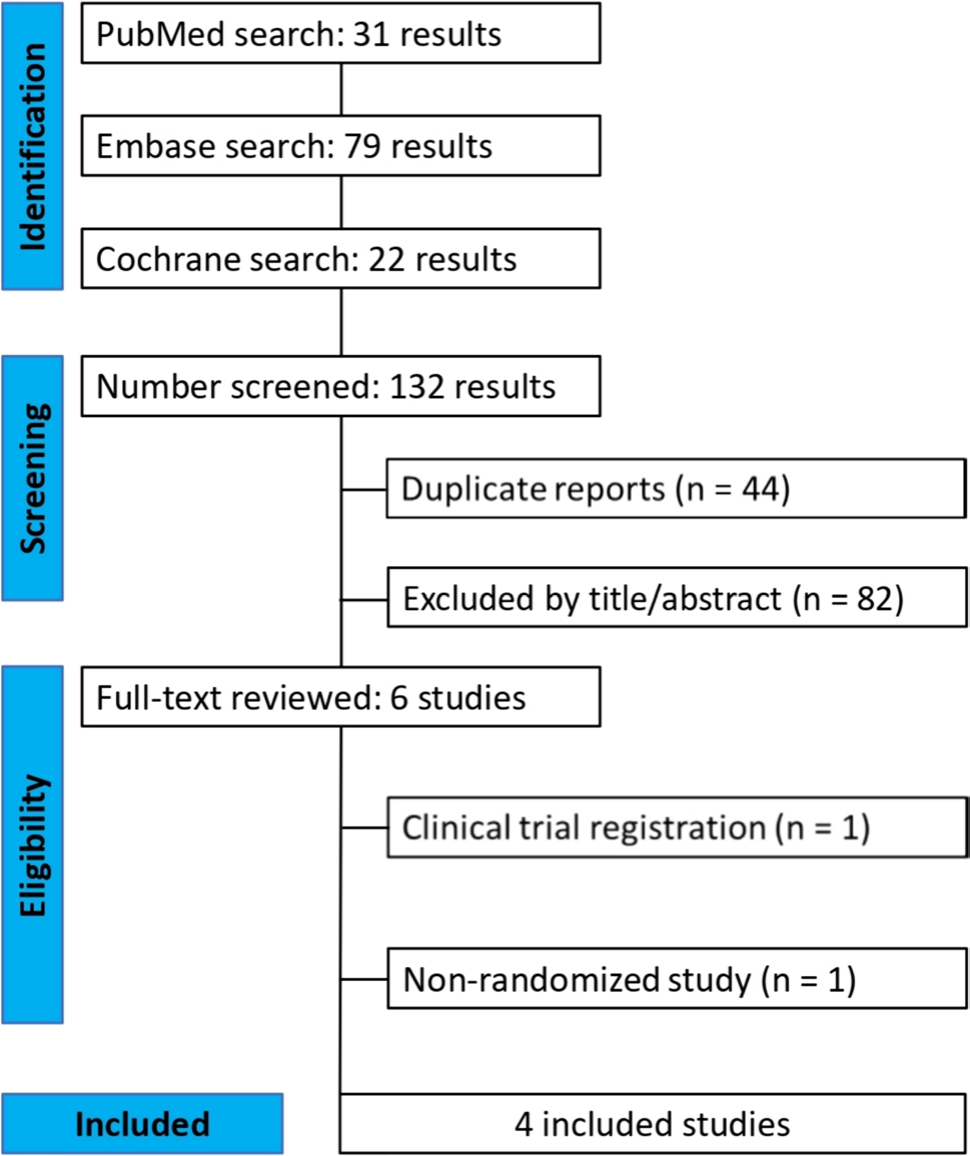
PRISMA flow diagram of study screening and selection

As shown in Fig. 1, the search strategy yielded 132 studies. After removing duplicate records and studies with an exclusion criterion based on title/abstract review, six remained and were thoroughly reviewed for inclusion and exclusion criteria. Four RCTs [14–17] were included, with a total of 381 patients, of which 57% (*n* = 217) were male, with a mean age ranging from 7.86 to 9.28 years. The follow-up periods in the included studies varied: 24 weeks in two studies [14–16] and 52 weeks in one [17]. Of the 381 patients, 186 (48.8%) were randomized to receive LEV monotherapy, whereas the remaining patients received CBZ monotherapy. The main characteristics of the included studies are summarized in Table 1.

**Table 1:** Baseline Characteristics of Patients

Study	Year	Design	Country	Number of patients LEV/CBZ	Male (%) LEV/CBZ	Age (years) ^a^ LEV/CBZ	Initiation drug dose ^b^ LEV/CBZ	Target drug dose ^b^ LEV/CBZ	Follow-up (weeks) ^c^ LEV/CBZ
Jung et al. [17]	2015	RCT (multicenter, open-label, non-inferiority clinical trial)	South Korea	57/64	52/60	9.28 / 8.05	20/10	40/20	52
Akhondian et al. [16]	2020	RCT (monocenter, single-blinded)	Iran	23/25	56/60	7.32/7.89	10/5	30/15	24
Ahadi et al. [15]	2020	RCT (monocenter, open-label)	Iran	46/46	56/60	8.7/8.36	25–30/ 15–20	-/-	24
Montazerlotfelahi et al. [14]	2024	RCT (monocenter, double-blinded)	Iran	60/60	53/60	7.96/7.86	10/5	30/15	24

*Abbreviations*: *RCT* Randomized Controlled Trial, *LEV* Levetiracetam, *CBZ* Carbamazepine

^a^ Mean

^b^ Mg/kg/day

^c^ Mean ± SD

-/- No information available

### Primary outcome

**Fig. 2:**
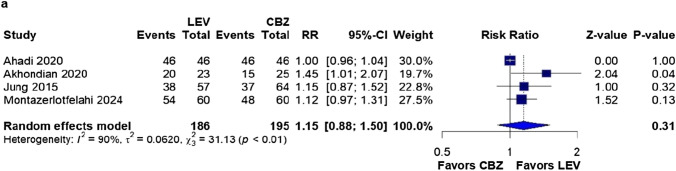
There was no significant difference in seizure freedom between patients undergoing monotherapy with LEV and those with CBZ

Seizure freedom was achieved in 158 (84.9%) of the 186 patients assigned to the LEV group, compared with 146 cases (74.9%) among the 195 children undergoing CBZ. We found no significant differences between the groups in this analysis (RR: 1.15; 95% CI 0.88–1.50; *p* = 0.31; I^2^ = 90%; Fig. 2).

### Secondary outcomes

**Fig. 3:**
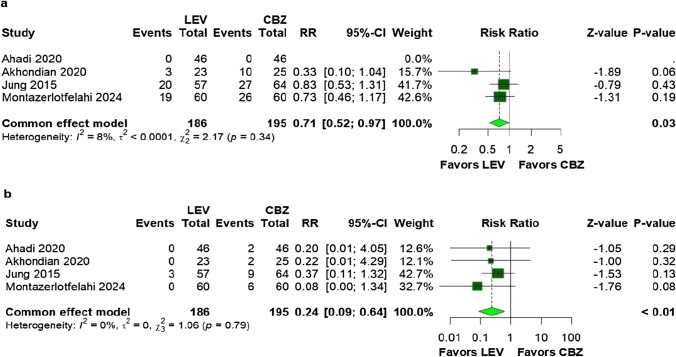
There were significant differences between patients undergoing monotherapy with LEV compared to CBZ for the following outcomes: **a** frequency of at least one seizure; **b** dermatologic adverse events

The frequency of at least one seizure (RR: 0.71; 95% CI 0.52–0.97; *p* = 0.03; I^2^ = 8%; Fig. 3a) and dermatologic adverse events (RR: 0.24; 95% CI 0.09–0.64; *p* < 0.01; I^2^ = 0%; Fig. 3b) were significantly lower among patients treated with LEV. Specifically, the LEV group had three cases (1.9%) of dermatologic adverse events compared to 19 cases (13.0%) in the CBZ group.

**Fig. 4:**
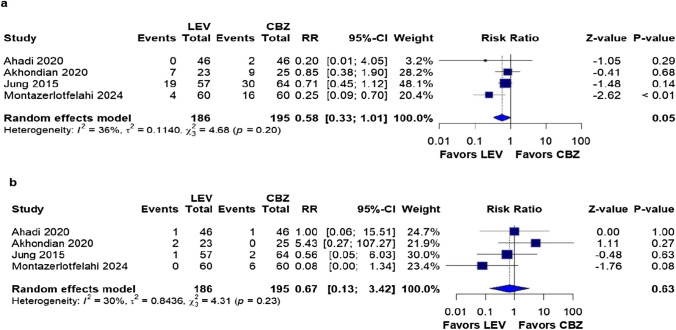
There were no significant differences between patients undergoing monotherapy with LEV or CBZ for the following outcomes: **a** any adverse events; **b** adverse events leading to treatment discontinuation

We found no significant differences in any adverse events (RR: 0.58; 95% CI 0.33–1.01; *p* = 0.05; I^2^ = 36%; Fig. 4a) or adverse events leading to treatment discontinuation outcomes (RR 0.67; 95% CI 0.13–3.42; *p* = 0.63; I^2^ = 30%; Fig. 4b).

### Sensitivity analyses

Sensitivity analyses were performed to address potential sources of heterogeneity and to assess the stability and robustness of the outcomes of seizure freedom, any adverse events, and adverse events leading to treatment discontinuation. Specifically, we utilized the Sidik-Jonkman-Hartung-Knapp method to estimate the between-study variance in the random effects model.

**Fig. 5:**
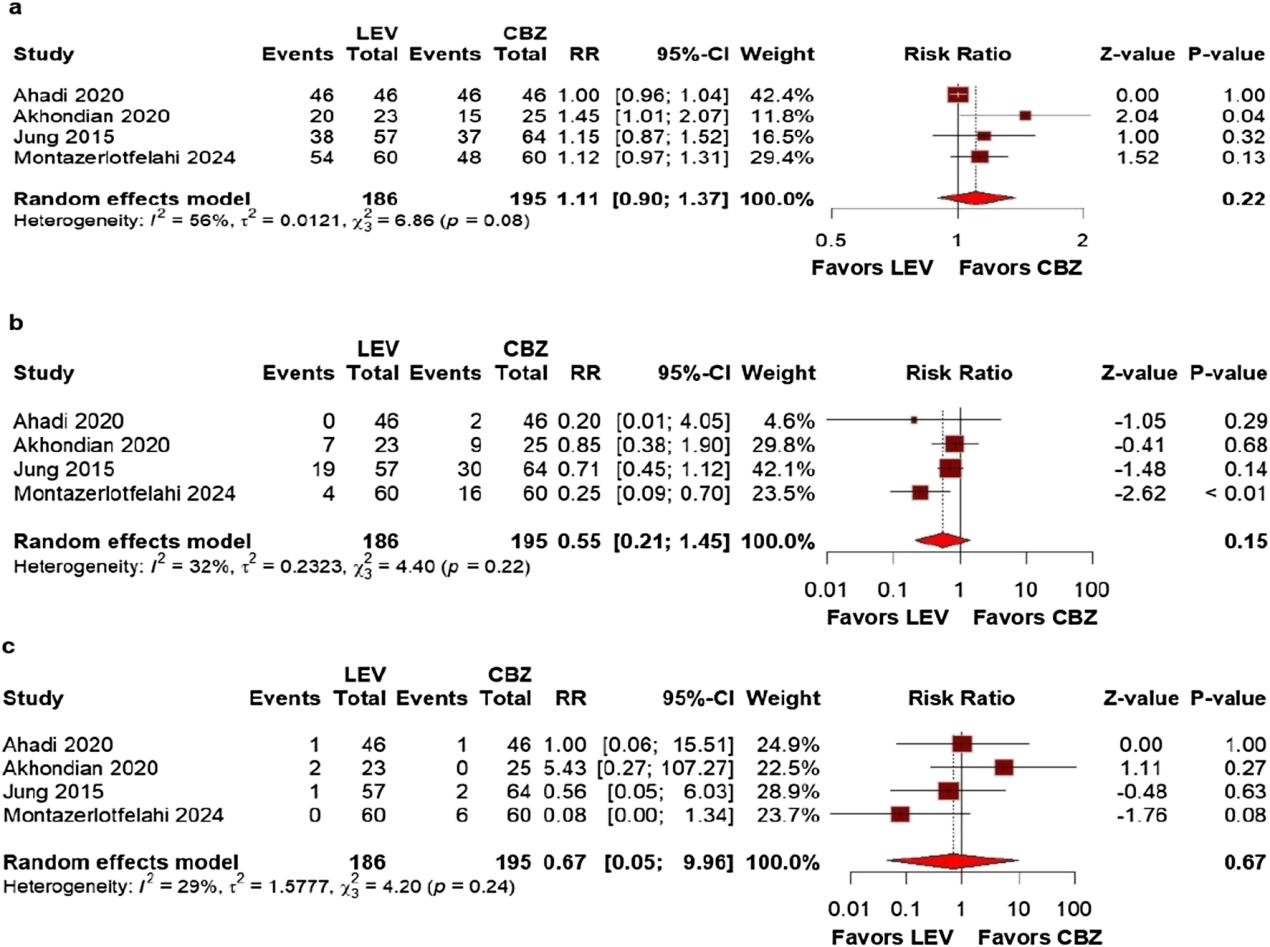
The sensitivity analyses confirmed the robustness of the results, with slightly adjusted but still non-significant effect estimates for the following outcomes: **a** seizure freedom; **b** any adverse events; and **c** adverse events leading to treatment discontinuation

Regarding seizure freedom, the results remained non-significant (RR: 1.11; 95% CI 0.90–1.37; *p* = 0.22; I^2^ = 56%; Fig. 5a). Similarly, there was no difference in the occurrence of any adverse events (RR: 0.55; 95% CI 0.21–1.45; *p* = 0.15; I^2^ = 32%; Fig. 5b), including those leading to treatment discontinuation (RR: 0.67; 95% CI 0.05–9.96; *p* = 0.67; I^2^ = 29%; Fig. 5c).

### Quality assessment

**Fig. 6:**
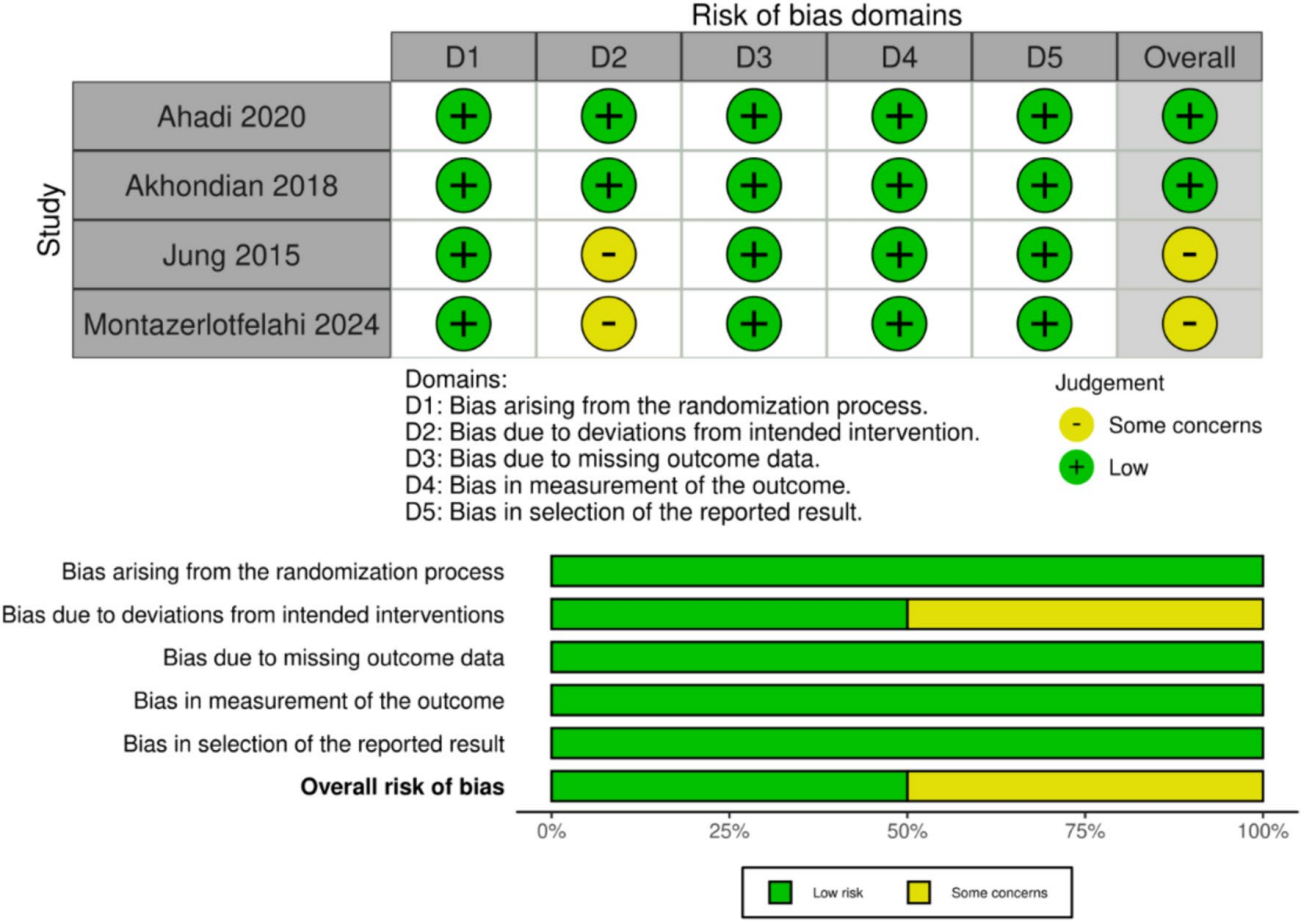
Risk of bias assessment

Figure 6 outlines the individual evaluations of each RCT in all five domains. Of all RCTs included, only two were deemed to have a low risk of bias [15, 16]. The two remaining studies were considered to have some concerns owing to uncertainty regarding the intended intervention analysis [14, 17].

According to the GRADE evaluation, the frequency of seizures and rate of dermatologic adverse events were deemed to have a high certainty of evidence. The rate of any adverse event and the rate of adverse events leading to treatment discontinuation were considered to have moderate certainty, downgraded due to moderate heterogeneity (I^2^ = 36% and I^2^ = 30%, respectively). Finally, the rate of seizure freedom was deemed to have low certainty, downgraded due to high heterogeneity (I^2^ = 90%) (Table 2).

**Table 2:** The GRADE quality of evidence

Certainty assessment							№ of patients		Effect		Certainty
№ of studies	Study design	Risk of bias	Inconsistency	Indirectness	Imprecision	Other considerations	Levetiracetam	Carbamazepine	Relative (95% CI)	Absolute (95% CI)	
The rate of seizure freedom											
4	RCT	serious	very serious (a)	not serious	not serious	none	158/186 (84.9%)	146/195 (74.9%)	RR 1.15 (0.88 to 1.50)	112 more per 1.000 (from 90 fewer to 374 more)	⨁⨁◯◯Low
The rate of any adverse events											
4	RCT	not serious	serious (b)	not serious	not serious	none	30/186 (16.1%)	57/195 (29.2%)	RR 0.58 (0.33 to 1.01)	123 fewer per 1.000 (from 196 fewer to 3 more)	⨁⨁⨁◯ Moderate
The rate of dermatologic adverse events											
4	RCT	serious	not serious	not serious	not serious	strong association	3/186 (1.6%)	19/195 (9.7%)	RR 0.24 (0.09 to 0.64)	74 fewer per 1.000 (from 89 fewer to 35 fewer)	⨁⨁⨁⨁ High
The rate of adverse events leading to treatment discontinuation											
4	RCT	not serious	serious (c)	not serious	not serious	none	4/186 (2.2%)	9/195 (4.6%)	RR 0.67 (0.13 to 3.42)	15 fewer per 1.000 (from 40 fewer to 112 more)	⨁⨁⨁◯Moderate
The frequency of seizures											
4	RCT	not serious	not serious	not serious	not serious	none	42/186 (22.6%)	63/195 (32.3%)	RR 0.71 (0.52 to 0.97)	94 fewer per 1.000 (from 155 fewer to 10 fewer)	⨁⨁⨁⨁ High

a. High heterogeneity (I^2^ = 90%)

b. Moderate heterogeneity (I^2^ = 36%)

c. Moderate heterogeneity (I^2^ = 30%)

**Fig. 7:**
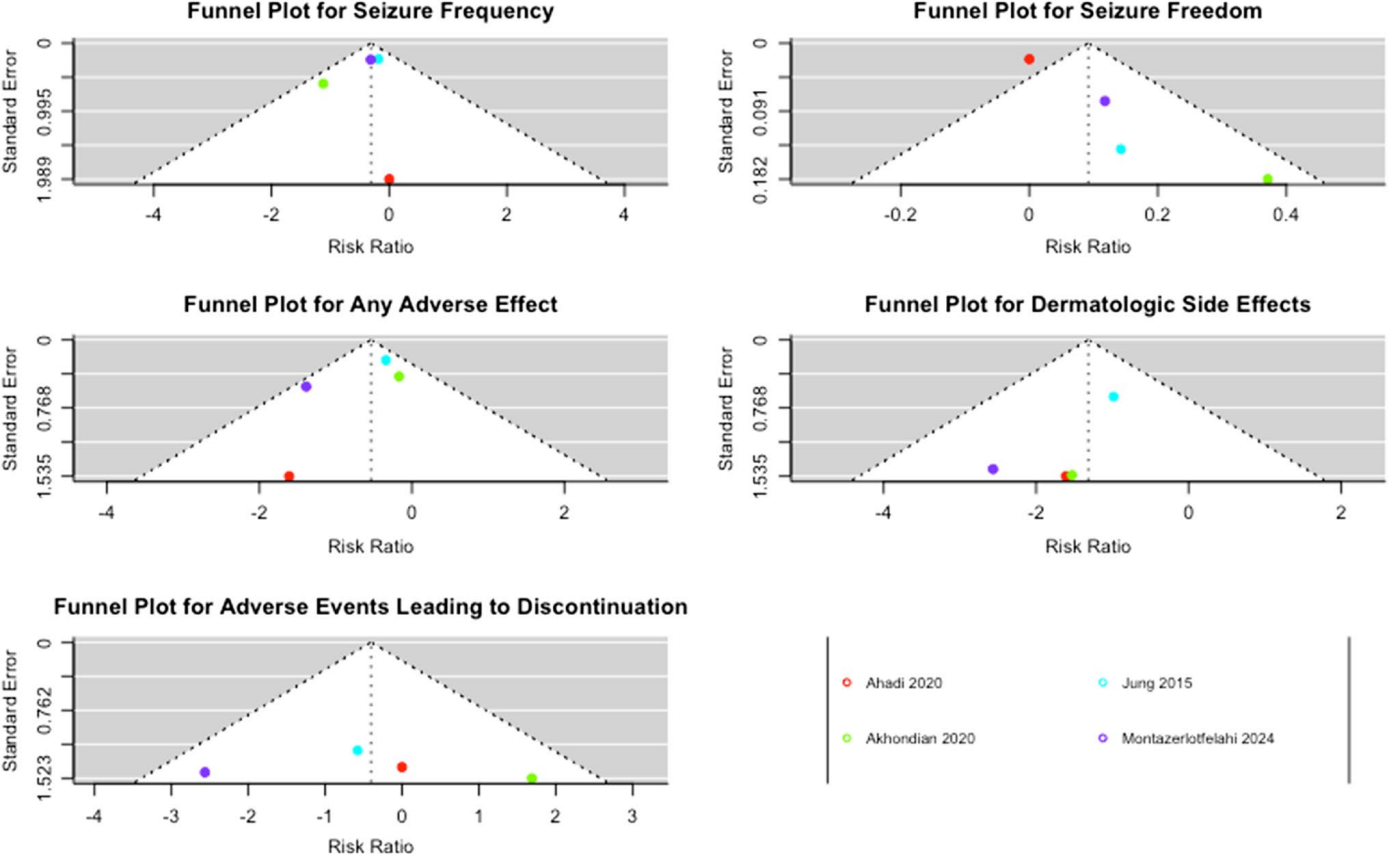
Funnel plots for each outcome showed symmetrical distributions around the pooled effect size, indicating low publication bias and consistent findings across studies, except for seizure freedom and dermatologic side effects; with more studies on one side than on the other

The funnel plot for the frequency of at least one seizure showed a symmetrical distribution centralized around a RR of zero, indicating no publication bias and consistency among the studies. Most studies were within the expected range for seizure freedom, but one study was identified as an outlier, suggesting potential bias or significant difference. The plot for dermatologic adverse events showed asymmetry, indicating possible publication bias or heterogeneity among studies. The funnel plot for any adverse event showed a symmetrical distribution centered around zero, suggesting no significant publication bias, with consistent results across studies. The plot for adverse events leading to treatment discontinuation was also symmetrical and centered around a RR of zero, but with a wider spread, indicating variability in effect sizes among the studies (Fig. 7).

## Discussion

In this systematic review and meta-analysis of four RCTs including 381 patients, we compared LEV with CBZ monotherapy for the treatment of focal epilepsy in children. The main findings were as follows: The LEV group had a 29% relative reduction in the frequency of at least one seizure and a 76% reduction in dermatological adverse events compared to the CBZ group. However, there were no significant differences between the groups regarding seizure freedom, any adverse events, or adverse events leading to treatment discontinuation.

Regarding the different follow-up intervals among the included studies, the RCT with a period of 52 weeks showed no significant differences in its results compared to other articles with intervals of 24 weeks [17]. Therefore, it can be suggested that the effects of LEV and CBZ treatments remain consistent over time. However, studies with longer follow-up periods are needed to provide additional information on the long-term efficacy and safety of these treatments. Further studies with longer follow-up periods are required to confirm these findings and assess the stability of the results over time. Considering this, even if there are a large number of non-RCT studies, there are few comparative RCTs on this issue.

Furthermore, there were no significant differences between the groups regarding the absence of seizures, any adverse events, or adverse events that led to treatment discontinuation, which revealed that LEV and CBZ had a similar impact. In previous studies, it was observed that other adverse events, such as behavioral changes and psychotic reactions, were reversible after LEV discontinuation [20]; however, this situation was not addressed in the included studies. Another relevant factor in this sense is the clarification of the mechanism of action of LEV and its comparison with the mechanism of action of CBZ to analyze other possible impacts.

It is important to highlight that in one study, two cases that developed intense agitation when using LEV were excluded, and agitation was a relatively frequent side effect in this group, while drowsiness and impaired consciousness were more common in the group treated with CBZ. The response to therapy was superior in the group that used LEV than in the group treated with CBZ [16]. In contrast, our analysis demonstrated no significant differences in other adverse events between the two therapies.

Most people with epilepsy are treated with monotherapy, and the United Kingdom National Institute for Health Excellence (NICE) guidelines recommend CBZ or lamotrigine as first-line treatment for focal seizures in children and adults. One study concluded that CBZ and lamotrigine are the best treatment options, and LEV may be a suitable alternative for individuals with focal seizures [18]. Another study directly compared LEV and CBZ and concluded that LEV monotherapy could offer superior efficacy and a lower risk of adverse effects than CBZ monotherapy, representing an important monotherapy treatment option for non-lesional focal epilepsy [5], as shown in our meta-analysis.

Initially, LEV was approved as an adjunctive therapy for focal seizures in children aged 1 to 15 years [15]. Current literature demonstrates that switching from an adjunctive therapy regimen to a monotherapy regimen improves seizure frequency. In a systematic review that included 1763 patients aged 0 to 18 years, it was proposed that LEV, even as an adjunctive treatment, could significantly reduce seizure frequency and be well tolerated by users. Furthermore, lower doses of LEV, starting at 20 mg/kg per day, provide more efficient control of focal seizures [19].

Our systematic review and meta-analysis has some limitations. Owing to the limited number of studies, the sample size was small. Additionally, there was a predominance of studies from Iran, which may limit the generalizability of our findings. Although these limitations may restrict the robustness of the conclusions, this is the first meta-analysis on the efficacy and tolerability of LEV compared to CBZ in the treatment of PFE. Thus, to date, this represents the best available evidence on this topic. Additionally, the heterogeneity among the studies was significant for the three outcomes of our analysis. Nevertheless, we assessed this heterogeneity using sensitivity analyses and the results remained consistent. As with any meta-analysis, our study was subject to publication bias, which may have been mitigated by the exclusive use of RCTs, and the solid results of the funnel plots.

## Conclusion

LEV monotherapy is associated with a lower frequency of seizures and fewer dermatological adverse events than CBZ monotherapy in patients with PFE. However, no differences were observed between LEV and CBZ in terms of overall seizure freedom, any adverse events, or adverse events leading to treatment discontinuation. These findings suggest that while both LEV and CBZ are effective monotherapies, LEV may offer advantages in specific adverse event profiles and seizure management. Nonetheless, owing to the limited sample size, further RCTs are welcome.

## Supplementary Information

Below is the link to the electronic supplementary material.

Supplementary file1 (JPG 210 KB)

## Data Availability

No datasets were generated or analysed during the current study.

## Abbreviations

CI: Confidence interval
CBZ: Carbamazepine
GRADE: Grading of Recommendations, Assessment, Development and Evaluation
LEV: Levetiracetam
PFE: Pediatric focal epilepsy
PRISMA: Preferred Reporting Items for Systematic Reviews and Meta-Analysis
RCT: Randomized controlled trial(s)
RR: Risk ratio
RoB-2: Cochrane’s Risk of Bias 2 software
