# Supplementary figures and images for: Levetiracetam versus carbamazepine monotherapy in the management of pediatric focal epilepsy: A systematic review and meta-analysis of randomized controlled trials
Source: Eur J Pediatr. 2024 Sep 18;183(11):4623–33. doi: 10.1007/s00431-024-05768-0 (PMC11473619; doi:10.1007/s00431-024-05768-0)

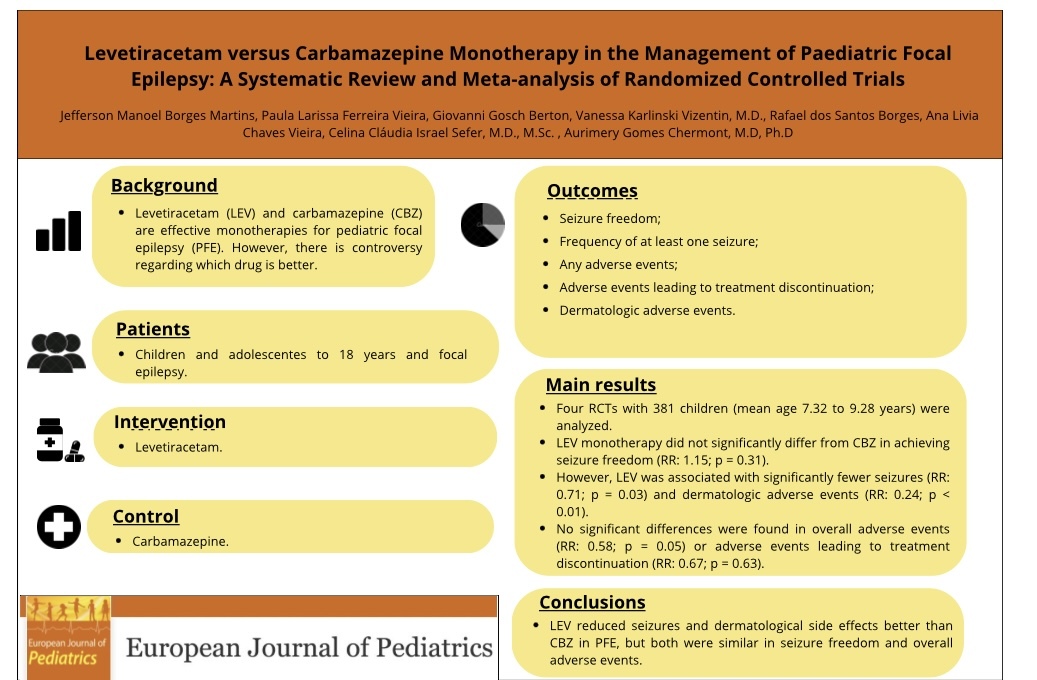

Supplement: Supplementary file 1 — Supplementary file1 (JPG 210 KB) [file 431_2024_5768_MOESM1_ESM.jpg]
